# Potential Therapies for Infectious Diseases Based on Targeting Immune Evasion Mechanisms That Pathogens Have in Common With Cancer Cells

**DOI:** 10.3389/fcimb.2019.00025

**Published:** 2019-02-12

**Authors:** Jodi Wong, Stephen Yiu Chuen Choi, Rongrong Liu, Eddie Xu, James Killam, Peter W. Gout, Yuzhuo Wang

**Affiliations:** ^1^Department of Experimental Therapeutics, BC Cancer Research Centre, Vancouver, BC, Canada; ^2^Vancouver Prostate Centre, Vancouver, BC, Canada; ^3^Department of Urologic Sciences, The University of British Columbia, Vancouver, BC, Canada; ^4^Department of Microbiology, School of Basic Medicine, Fourth Military Medical University, Xi'an, China

**Keywords:** immune evasion, pathogen, bacteria, endoparasite, virus, cancer, metabolic reprogramming, aerobic glycolysis

## Abstract

Many global infectious diseases are not well-controlled, underlining a critical need for new, more effective therapies. Pathogens and pathogen-infected host cells, like cancer cells, evade immune surveillance via immune evasion mechanisms. The present study indicates that pathogenic bacteria, endoparasites, and virus-infected host cells can have immune evasion mechanisms in common with cancers. These include entry into dormancy and metabolic reprogramming to aerobic glycolysis leading to excessive secretion of lactic acid and immobilization of local host immunity. The latter evasion tactic provides a therapeutic target for cancer, as shown by our recent finding that patient-derived cancer xenografts can be growth-arrested, without major host toxicity, by inhibiting their lactic acid secretion (as mediated by the MCT4 transporter)-with evidence of host immunity restoration. Accordingly, the multiplication of bacteria, endoparasites, and viruses that primarily depend on metabolic reprogramming to aerobic glycolysis for survival may be arrested using cancer treatment strategies that inhibit their lactic acid secretion. Immune evasion mechanisms shared by pathogens and cancer cells likely represent fundamental, evolutionarily-conserved mechanisms that may be particularly critical to their welfare. As such, their targeting may lead to novel therapies for infectious diseases.

## Introduction

Infectious diseases constitute a major health problem worldwide and impose a substantial burden on societies in human suffering and economic loss. While diseases like smallpox have essentially been eliminated, many other pathogen-induced diseases are not well-controlled and new infectious diseases are continually emerging, thus underlining a critical need for novel therapeutic targets leading to more effective therapies (Fonkwo, 2008).

The human body is constantly under attack by foreign microorganisms such as pathogenic bacteria, parasites, and viruses, i.e., pathogens whose invasion of the body and subsequent multiplication can lead to a wide variety of global infectious diseases. In defense of the body, invading pathogens are identified by the *innate immune system* as “non-self” via cell surface pattern recognition receptors (PRRs) on innate immune cells which recognize pathogen-associated molecular patterns (PAMPs) on the foreign microbes; the latter are then eliminated by, for example, macrophage-mediated phagocytosis or secretion of cytotoxins by NK cells (Chaplin, 2010; Vaure and Liu, 2014).

Recognition of foreign microbes by *adaptive immunity* is based on the ability of T- and B-lymphocytes to distinguish self from non-self-antigens via cell surface antigen-specific receptors, i.e., T-cell receptors (TCRs) and B-cell receptors (BCRs). Identification of pathogen-infected host cells is achieved by T cells via recognition of antigen fragments presented by major histocompatibility complex (MHC) molecules. Elimination of the intruders is accomplished via cytotoxic responses by CD8^+^ T cells and CD45^+^ lymphocytes, recruitment of neutrophils, monocytes, and mature B lymphocytes (mediated by CD4^+^ helper T cells), as well as antibody production by plasma cells.

In addition to foreign microorganisms, host cancer cells can be recognized by the immune system as changes in their cell surface structure render them foreign (Chaplin, 2010).

Despite vigorous immune surveillance by healthy, immunocompetent hosts, pathogens often manage to survive in the body. They accomplish this by employing a large variety of immune evasion mechanisms based on, for example, immobilizing immune responses or avoiding recognition by host immune cells (Finlay and McFadden, 2006; Reddick and Alto, 2014). Such survival tactics can also be used by cancer cells (Chaplin, 2010; Vinay et al., 2015). In the last decade it has become evident that metabolic reprogramming to aerobic glycolysis (the Warburg effect) is a key mechanism that most cancer types use to evade host immune surveillance, as the resulting increases in lactic acid levels and acidity of the tumor micro-environment immobilize host immune cell activity (Fischer et al., 2007; Choi et al., 2013; Brand et al., 2016). As recently shown, this immune evasion tactic provides a target for cancer therapy (Choi et al., 2016, 2018).

In the present study, we have compared immune evasion mechanisms of pathogens (i.e., bacteria, endoparasites, and virus-infected host cells) and human cancer cells in search of mechanisms common to both groups. Such shared tactics may represent fundamental, evolutionarily-conserved mechanisms of immune evasion, which could in turn be useful as targets for novel therapies of both cancer and infectious diseases.

## Immune Evasion Mechanisms Employed By Pathogens and Cancer Cells

A literature survey led to the following information.

### Pathogenic Bacteria

A small percentage of bacterial species can cause globally significant diseases such as tuberculosis and pneumonia. A typical immune response to pathogenic bacteria consists of opsonisation of the microbes followed by phagocytosis by host macrophages and fusion of the phagosomes with lysosomes. Intra-lysosomal acid hydrolases, reactive oxygen species (ROS) and nitric oxide ultimately kill the pathogens (van Kessel et al., 2014).

Bacteria have developed a number of immune evasion mechanisms and methods to manipulate host machinery to promote their survival and proliferation. They can avoid recognition by TLRs by masking their surface antigens with a carbohydrate capsule (Finlay and McFadden, 2006) or through alterations of cell surface lipids (Cambier et al., 2014). Some bacterial species are able to subvert the phagocytic process by secreting proteinaceous effectors into the host cell via their “type three secretion systems” (T3SS) (Quitard et al., 2006) to target host actin remodeling required for phagocytosis (Finlay and McFadden, 2006) and to promote entry into host cells (Betts et al., 2009; da Cunha et al., 2014). Other species manage to use the host intracellular environment for proliferation, with *Mycobacterium tuberculosis, Coxiella burnetii*, and *Listeria monocytogenes* residing in macrophages, *Salmonella enterica* in the intestinal epithelium, *Mycobacterium leprae* in connective and nerve tissues, and *Chlamydia trachomatis* in ocular and urogenital tissues (Betts et al., 2009; da Cunha et al., 2014). The MAPK/NFκB pro-inflammatory pathway can also be targeted by bacterial proteases to inhibit an inflammatory response, and TLR signaling can be modulated to secrete the immunosuppressive cytokine IL-10 (Finlay and McFadden, 2006).

When phagocytized, bacteria employ various strategies to survive. Commonly, they escape the phagosomal environment prior to its lysosome-mediated acidification: *Shigella* and *L. monocytogenes* escape by remodeling host actin and secreting phospholipases to cleave the phagosomal membrane (Small et al., 1994; Baxt et al., 2013). Some bacteria, such as *M. tuberculosis*, prevent the acidification of the phagosome by disrupting phagosome-lysosome fusion using the PtpA tyrosine phosphatase (Bach et al., 2008). Others exploit the phagosome to create a suitable microenvironment in which to proliferate: *Legionella pneumophila* creates a vacuolar environment specifically lacking MHC class II molecules, thereby protecting itself from innate immunity (Clemens and Horwitz, 1992), whereas *C. burnetii* requires an acidic environment for growth and virulence (Maurin et al., 1992).

In response to attack by the immune system, bacteria can enter a hardy, non-replicating state, termed dormancy, for protection (Rittershaus et al., 2013). Dormancy comes as two different types: (i) cellular quiescence, a period of reduced cellular growth that maintains a basal metabolism as observed for persistent *M. tuberculosis* (Betts et al., 2002) and (ii) true dormancy, a metabolically-arrested spore state promoting survival under adverse conditions, as exhibited by the difficult-to-treat *Clostridium* genus (Rittershaus et al., 2013). Cellular quiescence is achieved through stress-triggered expression of dormancy-related genes, e.g., activation of signaling system-related genes such as the type II toxin-antitoxin (TA) module and *tnaA*, a gene responsible for the conversion of tryptophan to indole, an intracellular signaling molecule responsible for mediating persistence (Wood et al., 2013; Pu et al., 2017).

Bacteria can also evade innate immunity by reprogramming their energy metabolisms. *Staphylococcus aureus*, in its hypoxic phagosomal environment, upregulates glycolysis and lactic acid fermentation in order to maintain a redox balance in response to immune-induced nitric oxide stress (Vitko et al., 2015). When glucose is abundant, *Escherichia coli* reprograms glycolysis toward the production of acetate in its exponential growth (Dittrich et al., 2005). Intra-macrophagic *Brucella abortus* induces a shift toward aerobic glycolysis in its host—a mechanism shown to be required for its survival (Czyz et al., 2017). The lactic acid and derivative short-chain fatty acids (SCFAs) produced by the bacteria are secreted in order to maintain an alkaline intracellular pH (Konings et al., 1997). The lactic acid and SCFAs may also act as immune-modulatory signaling molecules, as their production by the gut microbiota generates an immune-tolerogenic environment by promoting T_reg_ cell differentiation and inhibiting epithelial cell proliferation (Thangaraju et al., 2009; Iraporda et al., 2015; Asarat et al., 2016; Corrêa-Oliveira et al., 2016). Other evidence shows that volatile bacterial SCFAs can markedly inhibit T- and B-cell proliferation (Kurita-Ochiai et al., 1995).

### Pathogenic Endoparasites

Endoparasites, i.e., parasites living inside the human body, can be grouped into two main categories: single-celled protozoa and multicellular worms, helminths. Both require successful evasion of the host immune system for survival and reproduction (Cox, 2002).

Protozoa employ several adaptive mechanisms to avoid contact with immune cells, often seeking residence in immune privileged sites. *Plasmodium falciparum*, which causes malaria in humans, produces SPECT-1 and -2 proteins allowing it to evade humoral immunity while traveling to the liver where it can mature in a relative immune privileged environment (Patarroyo et al., 2011). It then travels in the peripheral blood and infects erythrocytes, which lack MHC I receptors, and hence cannot be targeted by cytotoxic immune cells (Gomes et al., 2016). *Trypanosoma Brucei*, causing African sleeping disease, proliferates in blood and lymph and invades the central nervous system—another immune privileged site (Masocha et al., 2007). Migrating helminths also evade immune attack by remaining in the central nervous system and liver (Pockros and Capozza, 2005). Polymorphism also plays a role in immune evasion by protozoa. *P. falciparum*, for example, go through multiple stages during their lifetime and alter their surface antigens after every stage, whereas *T. Brucei* survives through subsurface protein remodeling which is involved in signaling transitions during developmental stages of dormancy and disease progression (Batram et al., 2014). These polymorphic modifications downgrade the ability of B cells to make highly specific antibodies (Zambrano-Villa et al., 2002).

Another mechanism via which endoparasites survive the host defense is through immunomodulation; some protozoa are able to induce host T cell anergy through suppression of co-stimulators and cytokines (Zambrano-Villa et al., 2002; Rodrigues et al., 2014). *T. brucei* uses its “vector host” to its advantage, in which the saliva of the Tsetse fly transmitted along with the parasite contains a peptide which suppresses human host release of cytokines TNF-α, IFN-γ, IL-6, and IL-10 (Bai et al., 2015; Stijlemans et al., 2016). Helminths are able to survive in humans for many years due to their ability to secrete immunomodulatory products (Hewitson et al., 2009).

Entry into a dormant state is also a survival strategy of some endoparasites, with stress-induced dormancy allowing their persistence under adverse conditions. In particular, induction of cell cycle arrest by ring-stage *P. falciparum* has been observed following use of traditional anti-malarial artemisinin-based combination treatments (Witkowski et al., 2010) and amino acid starvation (McLean and Jacobs-Lorena, 2017). The dormant subpopulations upregulate key survival genes (e.g., genes encoding heat shock proteins), downregulate cell cycle regulators and DNA biosynthesis proteins, and sustain their energy needs through fatty acid synthesis and pyruvate metabolism. Importantly, drug-selected parasites were able to re-enter growth and development upon stress removal (Witkowski et al., 2010), accentuating an ever-pressing issue of parasitic persistence in disease treatment.

The metabolic profiles of endoparasites can also be modulated in response to variations in their environment and developmental stage. In proliferative stages, protozoa and helminths can display a preferred dependence on anaerobic glycolysis and lactic acid production for ATP generation. The intra-erythrocytic *Plasmodium* parasite displays deregulated glycolytic activity coupled to impaired mitochondrial metabolism, hypothesized to provide a distinct growth advantage during its proliferative stage (Salcedo-Sora et al., 2014). Parasitic helminths can proliferate in high oxygen environments with or without aerobic respiration, indicating a preference for aerobic glycolysis as well (Tielens, 1994).

### Pathogenic Viruses

Viruses can cause a wide variety of diseases such as influenza, HIV/AIDS and Ebola. They employ various strategies to evade and suppress the human host immune response. Primarily, viruses contain high sequence variability, which contributes to immune escape via interruption and alteration of antigen presentations from virus-infected cells (Balamurugan et al., 2017; Karlsson Hedestam et al., 2017). Influenza viruses, for example, evolve rapidly in a process called “antigenic drift,” gaining mutations in genes involved in antigen binding (Peacock et al., 2017; Wu and Wilson, 2017). Other mechanisms include T cell exhaustion leading to loss of cytotoxic function (Wieland et al., 2017) and reduced presentation of MHC class I and NKG2D molecules resulting in inadequate immune activation (Pymm et al., 2017; Schmiedel and Mandelboim, 2017).

Immune surveillance of viral pathogens is largely accomplished via intracellular sensors that detect viral RNA and DNA (Ma and Damania, 2016; Roy et al., 2016; Nerbøvik et al., 2017). Viruses can suppress the immune response by interfering with antiviral signaling pathways, particularly by modifying their nucleic acids, inhibiting proper expression of PRRs and their adaptors (Chan and Gack, 2016), and suppressing the production of antiviral cytokines (Zou et al., 2016). For example, the Seneca Valley virus can inhibit interferon responses by targeting host adaptors (Qian et al., 2017), and Dengue and West Nile viruses can block IFN-α/β receptors (Gack and Diamond, 2016). Furthermore, the role of gene-silencing microRNAs produced by human polyomaviruses has been found to downregulate expression of large T antigen, a target of antiviral immunity, thus mediating the immune escape and survival of the viruses (Martelli and Giannecchini, 2017).

Another immune evasion tactic of viruses consists of entering a dormant state within a host cell, with subsequent viral reactivation once cell immunity wanes. Cells infected with viruses that have become dormant upregulate expression of latency-associated genes, but not lytic viral genes, making immune detection of the viruses difficult (Phelan et al., 2017). Notably, HPV can, upon infection, either actively replicate or assume a state of dormancy (Hoppe-Seyler et al., 2017a,b). It has also been shown that migrating HIV-infected cells can reactivate following dormancy with differential response to drugs (Bohn-Wippert et al., 2017). Mechanisms underlying viral dormancy indicate epigenetic regulation in response to host-induced stressors; for example, binding of the innate immune sensor, IFI16, to the lytic gene promoter in KSHV-infected cells results in transcriptional repression of latent KSHV (Roy et al., 2016). Furthermore, post-translational histone modifications have also been associated with decreased antigen expression (Bloom et al., 2010; Arbuckle et al., 2017).

To optimize their environment for infection, viruses can alter many host cellular metabolic pathways; in particular, cells infected by actively replicating viruses exhibit cancer phenotypes such as aerobic glycolysis, upregulated glutaminolysis and fatty acid synthesis (Sanchez and Lagunoff, 2015). Viruses such as HBV, HIV, and Zika have been shown to dysregulate glycolysis by increasing GLUT1 expression, glucose influx and lactic acid production in their host cells (Masson et al., 2017). Moreover, an acidified microenvironment resulting from increased lactic acid secretion may contribute to virus-induced pathogenesis, as enveloped viruses' fusion to host cell membrane was observed to be more effective at a low pH (Desai et al., 2017). The fusion rate of the Ebola virus can also be increased by a brief exposure to an acidic environment (Markosyan et al., 2016).

### Cancer Cells

Although cancer cells originate from the human host, they can be recognized by the immune system as non-self due to changes in their cell surface structures, and must evade immune recognition to survive. They can accomplish this by masking their cancer-specific surface neoantigens to prevent recognition by NK and effector T cells, and by suppressing anticancer immune responses (Mohme et al., 2017). MHC class I molecules on tumor cell surfaces are often weakly expressed or lacking, thus precluding cytotoxic T-cell responses (Garrido et al., 1993). In another strategy, cancer cells incorporate host platelet-derived vesicles exhibiting MHC I molecules on their plasma membrane to disguise themselves with regular host antigens (Placke et al., 2012). Moreover, immune recognition was found to be reduced via downregulation of NKG2D, a transmembrane NK cell receptor complex in pancreatic, gastric, colorectal, and breast cancers (Wang et al., 2016; Bi and Tian, 2017), while the inhibitory PD-1/PD-L1 signaling pathway was upregulated (Munn and Bronte, 2016). Suppression of the immune system can also be achieved through activation of indoleamine 2,3-dioxygenase (IDO), an inducible enzyme that catalyzes the rate-limiting step in tryptophan catabolism. Elevated levels of IDO are produced by a variety of malignancies (Munn and Mellor, 2016) and result in IDO-induced degradation of tryptophan to kynurenine. This metabolite promotes T_reg_ differentiation (Chen et al., 2008), while tryptophan at diminished levels cannot activate mTORC1, an enzyme required for T-cell proliferation (Moon et al., 2015).

Cancer cells are also able to avoid immune surveillance by entering a latent state, termed tumor dormancy (Aguirre-Ghiso, 2007; Crea et al., 2015; Dong et al., 2017). Dormancy-capable cells, which express growth-inhibitory signals, self-impose a slow-cycling state and upregulate expression of pluripotency and self-renewal genes (Sosa et al., 2015; Malladi et al., 2016). Circulating tumor cells also adopt this dormant pro-survival phenotype (Mohme et al., 2017). Changes in the surrounding environment may awaken dormant cells into an immune-tolerogenic proliferative state.

A major immune evasion mechanism of cancers consists of reprogramming glucose metabolism to aerobic glycolysis (the Warburg effect) as distinct from the mitochondrial oxidative phosphorylation pathway used by normal cells (Warburg, 1956; Gatenby and Gillies, 2004; Choi et al., 2013; Agrawal and Rangarajan, 2015; Wong et al., 2015). The resulting excessive secretion of lactic acid by cancer cells, mediated by monocarboxylate transporters, MCT1 and MCT4, leads to abnormally high extracellular lactic acid levels and acidification of the tumor microenvironment; elevated MCT1 and MCT4 levels have been associated with poor cancer prognosis (Polanski et al., 2014; Marchiq and Pouysségur, 2016; Noble et al., 2017), with MCT4 being a main exporter of lactate from glycolytic tissues (Ullah et al., 2006). The highly elevated levels of extracellular lactic acid and decreased pH in the microenvironment act in combination to inhibit lactic acid secretion by T lymphocytes of both innate and adaptive immunity (Fischer et al., 2007; Calcinotto et al., 2012), thus reducing their glycolytic flux-dependent immune functions (Fischer et al., 2007; Calcinotto et al., 2012; Loftus and Finlay, 2016). The resultant intracellularly acidified immune cells undergo decreased metabolism and acidosis while the low-pH environment indirectly impairs cytokine production and secretion, both effects resulting in local immunosuppression (Fischer et al., 2007; Calcinotto et al., 2012; Chang et al., 2013; Tan et al., 2015; Paul et al., 2016; Bi and Tian, 2017). Aerobic glycolysis also has several proliferative advantages for cancers as the glycolytic intermediates can be used for biosynthesis of nucleotides and lipids essential for rapid cell proliferation (Lunt and Vander Heiden, 2011). The conversion of pyruvate to lactate is coupled to the reconversion of cofactor NADH to NAD^+^, which is essential for maintaining the glycolytic flux and intracellular redox status (Lunt and Vander Heiden, 2011). The acidic tumor microenvironment may also promote cancer growth by inducing epigenetic changes toward a proliferative stem cell-like profile in solid tumors (Som et al., 2016). Moreover, lactate itself can act as a signaling molecule responsible for immune modulation, mediating M2-polarization of tumor-associated macrophages, resulting in secretion of angiogenic factors and inducing tissue-remodeling inflammation (Shime et al., 2008; Colegio et al., 2014; Roszer, 2015).

## Discussion

As highlighted by this study, pathogenic bacteria, endoparasites and virus-infected host cells employ, like cancer cells, a large variety of mechanisms to evade immune surveillance. These mechanisms are diverse and multifaceted and, even within the four classes, various species may have their own unique methods of evading immune surveillance. As such, it is beyond the scope of this perspective article to elaborate on them. Further information can be obtained from the following literature references for pathogenic bacteria (Finlay and McFadden, 2006; Flannagan et al., 2015; Thammavongsa et al., 2015; Fisher et al., 2017; Karkhah et al., 2019), endoparasites (Cooper and Eleftherianos, 2016; Gomes et al., 2016; Nakada-Tsukui and Nozaki, 2016; Stijlemans et al., 2016; Wahlgren et al., 2017; Martínez-López et al., 2018), viruses (Finlay and McFadden, 2006; Chan and Gack, 2016; Agrawal et al., 2017; Felix and Savvides, 2017; Hsu, 2018; Soto et al., 2018), and cancer cells (Bhatia and Kumar, 2014; Vinay et al., 2015; Goodman et al., 2017; Bates et al., 2018).

**Figure 1:**
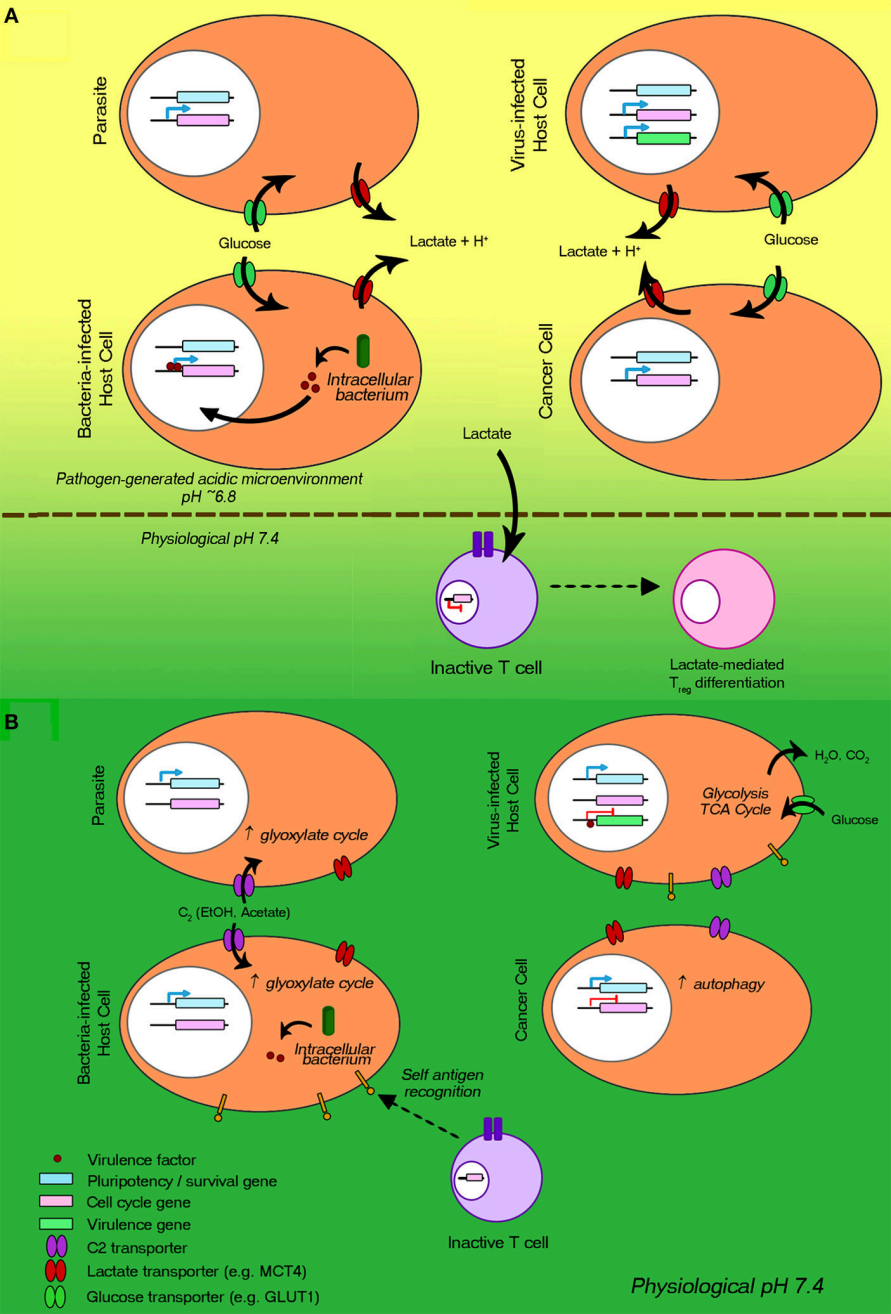
Metabolic reprogramming to aerobic glycolysis and entry into dormancy: two common immune evasion mechanisms employed by proliferating bacteria, endoparasites, viruses, and cancer cells. The host immune evasion/suppression processes are regulated via gene mutation and epigenetic reprogramming. **(A)** Processes involved in aerobic glycolysis are represented. Cell cycle genes are greatly upregulated. The reprogrammed metabolism leads to increases in (i) glucose uptake via glucose transporters (green) and (ii) lactic acid and short chain fatty acid secretion via monocarboxylate transporters (red), generating an acidic microenvironment. The lowered pH and increased lactate levels are immunosuppressive, e.g., inhibiting cytotoxic T cell activation. **(B)** Processes underlying entry into and maintenance of dormancy are illustrated. Pluripotency genes (blue) are expressed, while the expression of cell-cycle progression genes (pink) is arrested. Viral genes (green) of virus-infected cells are not expressed. Limited-nutrient metabolism states are induced (e.g., autophagy, glyoxylate cycle). T cells, recognizing self-antigens, are not activated.

Of special interest to us are the immune evasion mechanisms that the various pathogens share with cancer cells, as they likely represent fundamental, evolutionarily-conserved mechanisms that may be particularly critical to their welfare. Thus, such mechanisms could be useful as targets for novel therapies of both cancer and infectious diseases. In searching for such shared tactics two were found to be of paramount importance: (i) Metabolic reprogramming to aerobic glycolysis leading to excessive lactic acid secretion and consequent local host immunity suppression, a well-defined mechanism used by most human cancers (Gatenby and Gillies, 2004; Choi et al., 2013; Agrawal and Rangarajan, 2015) (Figure 1A). This tactic can also be used by pathogenic bacteria (Konings et al., 1997; Thangaraju et al., 2009; Iraporda et al., 2015; Vitko et al., 2015; Asarat et al., 2016; Corrêa-Oliveira et al., 2016; Czyz et al., 2017), endoparasites (Tielens, 1994; Salcedo-Sora et al., 2014), and virus-infected cells (Sanchez and Lagunoff, 2015; Markosyan et al., 2016; Desai et al., 2017; Masson et al., 2017). (ii) Entry into dormancy of pathogens or pathogen-infected host cells (Figure 1B), a less defined mechanism to avoid recognition by the immune system (Aguirre-Ghiso, 2007; Crea et al., 2015; Dong et al., 2017). It can be used by pathogenic bacteria (Betts et al., 2002; Rittershaus et al., 2013; Wood et al., 2013; Pu et al., 2017), endoparasites (Witkowski et al., 2010; McLean and Jacobs-Lorena, 2017), and virus-infected cells (Bohn-Wippert et al., 2017; Hoppe-Seyler et al., 2017a,b; Phelan et al., 2017).

Excessive secretion of lactic acid by cancer cells due to metabolic reprogramming to aerobic glycolysis provides a new potential target for cancer therapy. This has been demonstrated by our recent discovery that treatment of advanced prostate cancer cells with antisense oligonucleotides (ASOs) targeting MCT4-mediated lactic acid secretion led to a marked reduction in MCT4 expression and lactic acid secretion, increased intracellular lactic acid levels, and marked reductions in aerobic glycolysis, cell proliferation, cell migration, and tissue invasion (Choi et al., 2016, 2018). Treatment of PC-3 tumor-bearing nude mice with the anti-MCT4 ASOs markedly inhibited tumor growth without inducing major host toxicity (Choi et al., 2016; Choi, 2017). In addition, a limited study of the effect of MCT4-targeting on restoration of host immunity was carried out using clinically relevant, first-generation subrenal capsule xenograft models of patient-derived advanced prostate cancer. Such first-generation models lack systemic functional host immunity, but exhibit viable patient tumor-associated immune cells (Dong et al., 2017). Importantly, treatment of these models with anti-MCT4 ASOs led to an increase in the number of human CD45^+^ lymphocytes and CD8^+^ cytotoxic T cells, indicative of restoration of the host anticancer immunity (Choi, 2017). These findings raise the possibility that pathogens, which use metabolic reprogramming to aerobic glycolysis as a main immune evasion tactic, can also benefit from therapy based on inhibition of lactic acid secretion, particularly as the mechanism underlying transporter-mediated lactic acid secretion is relatively simple.

Although we here have outlined our perspective regarding potential therapeutic intervention resulting from inhibiting lactic acid secretion, we realize that possible approaches to reversing immune evasion are as vast and varied as the mechanisms themselves (Beatty and Gladney, 2015; Spranger and Gajewski, 2018). Alternative methods of reactivating the anti-cancer immune response are being actively investigated, the most successful of which involve neutralizing antibodies targeting immune checkpoint inhibitors such as PD-1 and CTLA-4 (Alsaab et al., 2017; Sharpe and Pauken, 2018; Tang et al., 2018). Furthermore, multiple components within a particular key pathway can also be considered therapeutic targets. Beyond MCT4, additional transporters as well as enzymes associated with aerobic glycolysis are also considered candidates for therapeutic inhibition of cancer (Tennant et al., 2010; Cairns et al., 2011; Zhao et al., 2013). Taken together, strategies of reactivating anti-cancer immunity have recently been shown to be successful across a number of cancer types (Goodman et al., 2017; Cascone et al., 2018; Seidel et al., 2018), offering evidence both scientifically and clinically that immunomodulation can translate into effective therapies. Similar considerations may apply to pathogen-induced diseases.

Finally, an additional aspect worth exploring as a potential therapeutic approach would be the use of combination therapies targeting multiple biological phenomena as surveyed here. In particular, targeting the dormant cell/organism population in conjunction with the actively proliferating population could offer enhanced efficacy. Given the importance of aerobic glycolysis to rapid cellular proliferation and the significance of cells entering dormancy to avoid immune detection, a combined effort to inhibit both lactic acid secretion (e.g., by MCT4 inhibition as mentioned above) and the unique metabolic properties of dormant cells could eliminate a greater proportion of cancers and pathogenic microorganisms. While mechanisms underlying cellular entry into dormancy remain to be more fully elucidated, preliminary investigations in our laboratory have uncovered changes in amino acid metabolism as a potentially unique metabolic profile in dormant cancer cells post-therapy (Nabavi et al., 2017). As such, targeting the metabolic rewiring that occurs within the dormant state could help eliminate even difficult-to-target residual cells.

## Conclusion

This study indicates that pathogenic bacteria, endoparasites, virus-infected host cells, and human cancer cells can share immune evasion mechanisms, i.e., (i) metabolic reprogramming to aerobic glycolysis leading to excessive lactic acid secretion and host immunity suppression and (ii) entry into dormancy. Targeting fundamental, evolutionarily-conserved mechanisms of immune evasion could be particularly effective for treatments of both cancer and multiple infectious diseases.

## Author Contributions

YW initiated the topic in July 2016. JW, SC, and PG organized the manuscript. RL and EX provided published evidence. JW, SC, JK, PG, and YW were involved in the further development of the ideas and the writing of the paper. PG finalized the write-up.

### Conflict of Interest Statement

The authors declare that the research was conducted in the absence of any commercial or financial relationships that could be construed as a potential conflict of interest.
